# Tree pollen allergy in Southwestern Ontario

**DOI:** 10.1186/1710-1492-8-S1-A11

**Published:** 2012-11-02

**Authors:** Gina Tsai, James Anderson, D William Moote

**Affiliations:** 1 Schulich School of Medicine, Western University, London, Ontario, Canada; 2 Environmental Allergy Assays, London, Ontario, Canada

## Background

Cross-reactivity among tree pollens is not as pronounced as that among grass or ragweed pollens. Seasonal pollen counts found in popular media only report selected tree pollen counts and may not reflect locally prevalent pollens.

## Methods

Atmospheric pollen in London, Ontario was collected with a Burkard air sampler from 1999-2009. Pollen was identified with light microscopy. The average monthly pollen count as well as total was calculated from January 1999 to December 2009. The local results were compared with those reported on the Weather Network website.

## Results

**Figure 1:**
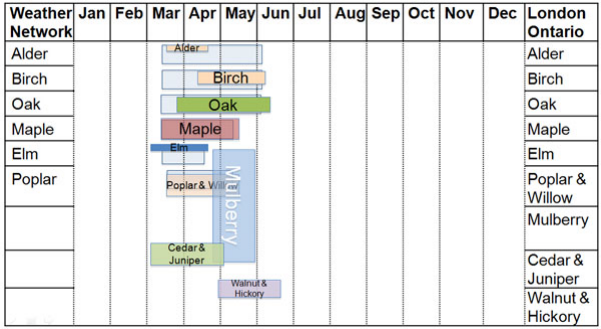
Transparent grey color represents the Weather Network pollen counts. Local counts are in color. The width of the bar represents the season, while the height represents quantity of the individual pollens. Note the small quantities of alder, birch and elm. Considerable mulberry, walnut/hickory and cedar/juniper present in our local counts were not reported by the Weather Network.

The Weather Network website only reports six tree pollens: alder, birch, oak, maple, elm and poplar, and do not consider regional variability in the seasons. In general, our seasons and pollen counts are in agreement with the Weather Network in terms of oak, maple and poplar pollens. However, local pollen counts detected lower quantities and shorter seasons for alder, birch and elm pollens. Large quantities of mulberry, cedar/juniper and walnut/hickory pollens were found in our local atmosphere, and not reported by the Weather Network. (see Figure 1)

## Conclusions

Local pollen counts may differ from those reported in popular media. The clinical relevance of the difference is not yet known. Knowledge of the local plant taxonomy and allergen cross-reactivity is important in selecting clinically relevant pollens for testing and immunotherapy, especially considering pollens that do not cross-react antigenically with those in the standard tree “mix”.

